# Molecular characteristics and antibiotic resistance mechanisms of clindamycin-resistant *Streptococcus agalactiae* isolates in China

**DOI:** 10.3389/fmicb.2023.1138039

**Published:** 2023-03-01

**Authors:** Zeliang Liu, Xueqi Jiang, Jie Li, Wenjing Ji, Haijian Zhou, Xinyi Gong, Beibei Miao, Shuang Meng, Like Duan, Qiyuan Shi, Xiao Han, Pengfang Gao, Chienyi Chang, Aiying Dong, Juan Li

**Affiliations:** ^1^Affiliated Hospital of North China University of Science and Technology, Tangshan, Hebei, China; ^2^State Key Laboratory for Infectious Disease Prevention and Control, Chinese Center for Disease Control and Prevention, National Institute for Communicable Disease Control and Prevention, Beijing, China; ^3^Department of Pharmacy Administration and Clinical Pharmacy, School of Pharmacy, Xi’an Jiaotong University, Xi’an, China; ^4^Faculty of Medical Sciences, School of Dental Sciences, Newcastle University, Newcastle upon Tyne, United Kingdom

**Keywords:** *Streptococcus agalactiae*, antibiotic sensitivity, whole genome sequencing, molecular type, virulence factor

## Abstract

*Streptococcus agalactiae* (Group B *Streptococcus*, GBS) is a major cause of neonatal infections with high morbidity and mortality, and clindamycin is the main antibiotic used to treat GBS infections in patients allergic to penicillin. We aimed to analyse the antibiotic sensitivity, sequence types, serotypes, virulence factors, and antibiotic resistance mechanisms of clinically isolated clindamycin-resistant *S. agalactiae* and provide basic data for the treatment, prevention, and control of clinical infection of *S. agalactiae*. A total of 110 strains of clindamycin-resistant *S. agalactiae* were collected from two tertiary hospitals in Hebei, China. We performed antibiotic sensitivity tests for 11 antibiotics on these strains and whole-genome sequencing analysis. All the strains were susceptible to penicillin, ampicillin, linezolid, vancomycin, tigecycline, and quinupristin–dalfopristin. Resistance to erythromycin, levofloxacin, tetracycline, and chloramphenicol were also observed. Genome sequence analysis revealed that all strains belonged to 12 sequence types (STs) related to six cloning complexes (CCs), namely CC10, CC19, CC23, CC651, CC1, and CC17. Five serotypes were identified, including IA, IB, II, III, and V. The most prominent resistance genes were *mreA* (100%) and *ermB* (81.8%). Furthermore, *cfb*, *cylE*, *pavA* and the gene cluster related to the pili were 100% present in all strains, followed by *lmb* (95.5%) and *srr1* (67.2%). This study found that clindamycin-resistant *S. agalactiae* showed polymorphisms in molecular types and serotypes. Furthermore, multiple virulence factor genes have been identified in their genomes.

## Introduction

*Streptococcus agalactiae* (Group B Streptococcus; Lancefield, 1933) is usually found in the digestive and urogenital tracts of pregnant women and adults. It can cause invasive infections in pregnant women, including bloodstream infection, meningitis, osteomyelitis, and endocarditis, can infect elderly patients and non-pregnant adults, and cause diseases such as bacteraemia, pneumonia, urinary tract infection, and skin/soft tissue infections (Raabe and Shane, 2019). In newborns, it is one of the main causes of neonatal morbidity and mortality worldwide, usually causing pneumonia, meningitis, or sepsis in newborns (Madrid et al., 2017). Maternal vertical infection is the main route of transmission for neonatal infections. *S. agalactiae* can cause foetal infection in the uterus or lead to neonatal infection through direct contact or inhalation at birth (ACOG, 2020). A study in 2015 showed that more than 300,000 newborn infections were caused by *S. agalactiae* worldwide, and 3.5 million premature infants were attributed to *S. agalactiae* infection (Seale et al., 2017). Another study revealed that approximately 19.7 million pregnant women had *S. agalactiae* colonisation, and nearly 400,000 newborns suffered from invasive diseases caused by *S. agalactiae* in 2020 (Goncalves et al., 2022). Furthermore, adults suffering from chronic diseases, such as diabetes, cancer, and HIV, also have a greatly increased infection rate of *S. agalactiae* (Francois et al., 2019; Slotved and Hoffmann, 2020; Graux et al., 2021), which causes endocarditis, pneumonia, bacteraemia, and urinary tract infection (Skoff et al., 2009; Ballard et al., 2016).

Clindamycin and erythromycin are two of the most important second-line antibiotics for the treatment of *S. agalactiae* infections, especially in penicillin-allergic patients. However, because erythromycin cannot pass through the placenta, and resistance is common, the importance of clindamycin was gained more attention (Bulska et al., 2015). Based on antibiotic susceptibility to clindamycin and erythromycin, the strain-resistance phenotype can be divided into four types: M phenotype (erythromycin resistant and clindamycin sensitive), L phenotype (erythromycin sensitive and clindamycin resistant), constitutive MLSb phenotype (cMLSb, concurrent erythromycin and clindamycin resistant), and inducible MLSb phenotype (iMLSb, erythromycin resistance induces clindamycin resistant; Hayes et al., 2020). According to the China Bacterial Drug Resistance Monitoring Network (CHINET), the resistance rates of *S. agalactiae* to clindamycin and erythromycin were as high as 59.7 and 74.5%, respectively, among 5,052 clinical strains between January and June 2022. The prevalence of *S. agalactiae* with MLSb phenotypes is increasing, and resistance mechanisms to clindamycin and erythromycin are multitudinous, including methyltransferases encoded by *erm* family genes, as well as *mef* and *mre* family genes encoding antibiotic efflux pumps (Gizachew et al., 2019; Meehan et al., 2021; Sapugahawatte et al., 2022). Fluoroquinolones are one of the important antibiotic classes for the treatment of *S. agalactiae* infection in adults, and mutations in *gyrA* and *parC*, encoding for DNA gyrase subunit and topoisomerase IV, respectively, reduce fluoroquinolone binding to their DNA targets, leading to enhanced tolerance to quinolones (Arias et al., 2019). The *tet* family genes that mediate drug efflux and protect ribosome targets are the main resistance mechanisms of *S. agalactiae* to tetracyclines; the common genes *tetA*, *tetK*, and *tetL* encode efflux proteins, while the prevalent genes *tetM*, *tetO*, and *tetS* encode ribosomal protection proteins (Haenni et al., 2018; Haimbodi et al., 2021).

The capsule, pili, and surface proteins of *S. agalactiae*, an opportunistic commensal bacterium that colonises humans, also play vital roles as virulence factors in bacteria-host interactions and pathogenicity. The capsular polysaccharides (CPS) of *S. agalactiae* can be divided into ten serotypes (IA, IB and II–IX), with a small proportion described as non-typeable (NT). The main serotypes associated with human pathogenesis are IA, IB, II, III, and V (Russell et al., 2017; Francois et al., 2019; Zhu et al., 2020), and the frequency of detecting serotypes varies based on geographical dispersion and other unknown factors. For example, serotypes III, V, and VI were dominant in Sri Lanka (Sapugahawatte et al., 2022), the frequency of serotype IA was higher than that of other serotypes in Brazil (Do et al., 2019), and serotype III was dominant in China (Cheng et al., 2020). Virulence factors include the α protein family (Alpha, Rib, Alp1 and Alp2/3; Furfaro et al., 2018; Paoletti and Kasper, 2019), serine-rich repeat protein (Srr; Seo et al., 2013; Lannes-Costa et al., 2021), hypervirulent GBS adhesin (HvgA; Tazi et al., 2010), laminin-binding protein (Lmb; Sridharan et al., 2019), Christie–Atkins–Munch–Petersen (CAMP) factor (Lang and Palmer, 2003), and pili (de Figueiredo et al., 2021a), all of which play important roles in infection type and severity. HvgA was detected in CC17 and CC23 strains and is the most virulent factor for invasive infection in newborns (Tazi et al., 2010; McGee et al., 2021).

The high rate of clindamycin-resistant *S. agalactiae* in China causes big challenges to the treatment of patients with severe penicillin allergy. However, little is known about the molecular characteristics, virulence factors, or resistance mechanisms of clindamycin-resistant strains. The aim of this study was to explore the clonal complexes distribution, virulence factors, and antibiotic resistance mechanisms through antibiotic sensitivity detection and whole genome sequence analysis of clinical clindamycin-resistant *S. agalactiae*, as well as to provide evidence for the precise treatment and control of *S. agalactiae* infection.

## Materials and methods

### Ethics statement

Strains were collected from patients with consent. This study was reviewed and approved by the ethics committee of the National Institute for Communicable Disease Control and Prevention, China CDC, in accordance with the medical research regulations of the Ministry of Health, China. The present study was conducted in China.

### Strain collection and identification

*S. agalactiae* strains isolated and preserved between May 2021 and December 2021 from the Affiliated Hospital of the North China University of Science and Technology and Tangshan Maternal and Child Health Hospital were collected. Duplicate strains from the same patients were eliminated. The strains were identified using Gram staining, colony morphology, and the CAMP test, and further identified using the Vitek 2 Compact strain identification antibiotic sensitivity analysis system. The disk diffusion method (Kirby-Bauer) was performed to screen for clindamycin-resistant strains.

### Detection of antibiotic sensitivity

BD Phoenix^™^ M50 was used to test the susceptibility profile against 11 antibiotics, including penicillin, ampicillin, linezolid, vancomycin, tigecycline, quinupristin/dalfopristin, clindamycin, erythromycin, levofloxacin, tetracycline, and chloramphenicol. *Streptococcus pneumoniae* (ATCC 49619) was used as a quality control strain. According to the recommendations and interpretation of the Clinical Laboratory Standards Institute (CLSI), strains resistant to erythromycin but sensitive to clindamycin were determined as MLSb resistance phenotype using the D-test with erythromycin (15 μg) and clindamycin (2 μg) from Oxoid Limited. If the inhibition zone surrounding the clindamycin disk adjacent to the erythromycin disk appeared truncated (in the shape of a “D”), the D test is positive, and the iMLSb phenotype is determined. Antibiotic sensitivity interpretation was performed based on the CLSI-M100-S23 from CLSI.

### Whole-genome sequencing

Pure cultures of *S. agalactiae* were cultivated for 18–24 h on Colombian agar with 5% sheep blood, and genomic DNA was extracted using the Wizard Genomic DNA Purification Kit (Promega, United States). The purified DNA was then sent to the MIGIGENE company for gene library construction and whole-genome sequencing (WGS) on the Illumina HiSeq 2000. The *de novo* genome was assembled from Illumina data using the SPAdes (v3.13.1) software.

### Multilocus sequence typing (MLST)

The specific sequence types (STs) and clonal complexes (CCs) were determined based on the gene sequences of the seven housekeeping genes, namely *adhP*, *pheS*, *atr*, *glnA*, *sdhA*, *glcK*, and *tkt* (Jones et al., 2003). The genome sequence was assembled and uploaded to a public database for molecular typing and microbial genome diversity to identify the ST and CC.^1^ If no strain was assigned to the corresponding ST, the genome file was uploaded to the webmaster to determine the new ST number and was added to the existing database. The MLST minimum spanning tree was constructed using the Bionumerics software (Applied Maths, Belgium).

### Data analysis

The resistance genes and virulence factors were determined based on the Center for Genomic Epidemiology database (Zankari et al., 2012),^2^ comprehensive antibiotic resistance database (CARD; Jia et al., 2017), and virulence factor database (VFDB; Liu et al., 2019), with a similarity threshold of > 90% and a coverage threshold of 60% compared to the reference sequences in the database. A previous study (McGee et al., 2021) on serotype and virulence factor gene analysis has been referred. To determine whether the changes in antibiotic resistance genes and virulence factors observed between CCs were the result of chance, a statistical comparison was conducted using the Chi-square test, which was statistically significant at *p* < 0.05.

### Data availability

The complete genomic sequences of all strains in our study were deposited at the China National Microbiology Data Center (NMDC, https://nmdc.cn/en) under BioProject Number 10018268.

## Results

### Strain characteristics

A total of 110 clindamycin-resistant *S. agalactiae* strains were collected in this study: 83 from routine maternal screening in late pregnancy, 4 from neonates, and 23 from non-pregnant adults. Four neonates had symptoms of infection, and four *S. agalactiae* strains were isolated from each of the four neonates, of which two were isolated from the external auditory canal, one from sputum, and one from blood. The age range of the 23 non-pregnant adults was 27–92 years, with a male-to-female ratio of 8:15; eight strains were isolated from wound secretions, seven from vaginal secretions, four from urine, two from sputum, and one each from blood and pus (Supplementary Table S1).

### Antimicrobial susceptibility profiles

**Figure 1:**
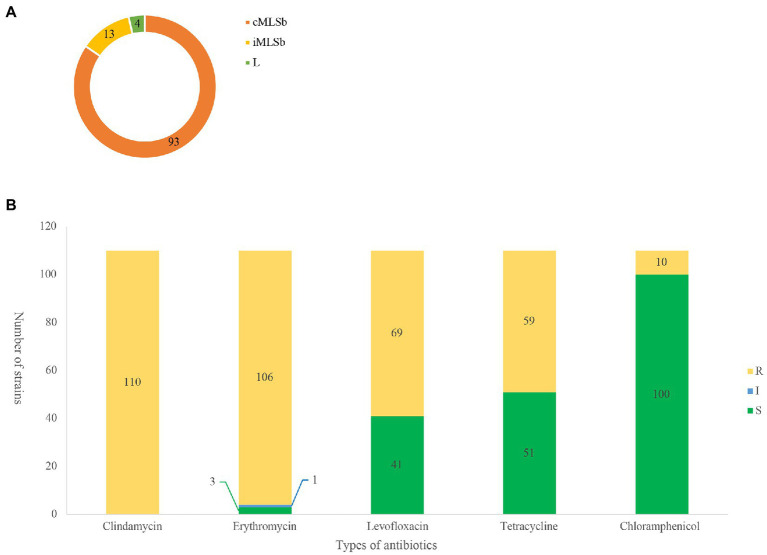
MLSb phenotypes and antibiotic susceptibility. Numbers of the different colours represent the number of strains (*n* = 110). **(A)**. Distribution of clindamycin/erythromycin resistant phenotypes in 110 clindamycin-resistant *Streptococcus agalactiae* strains. (**B)**. Antibiotic resistant profiles of 110 clindamycin-resistant *S. agalactiae* strains. R, resistance; I, intermediate; S, sensitive.

Among the 110 strains, M-type strain was not observed, while the predominant phenotype was cMLSb (84.5%, 93/110), followed by iMLSb (11.8%, 13/110; Figure 1A). All the strains were susceptible to penicillin, ampicillin, linezolid, vancomycin, tigecycline, and quinuptin/dapfamptin. In addition to clindamycin resistance, the strains were resistant to other antimicrobials: 96.3% (106/110) to erythromycin, 62.7% (69/110) to levofloxacin, 53.6% (59/110) to tetracycline, and 9.0% (10/110) to chloramphenicol (Figure 1B). We found six resistance patterns among the 110 strains: clindamycin-erythromycin-levofloxacin resistance accounted for 46.3% (51/110), followed by clindamycin–erythromycin–tetracycline resistance at 32.7% (36/110), as detailed in Table 1.

**Table 1:** Distribution of the resistance patterns of 110 clindamycin-resistant *Streptococcus agalactiae* strains.

Pattern	Antibiotics resistance pattern (Antibiogram)	No. of antibiotics to which strains were resistant	No. of strains (rate)
1	CLI-TE	2	3 (2.7%)
2	CLI-ERY-TE	3	36 (32.7%)
3	CLI-ERY-LEV	3	51 (46.4%)
4	CLI-ERY-TE-LEV	4	10 (9.1%)
5	CLI-ERY-TE-CHL	4	2 (1.8%)
6	CLI-ERY-TE-LEV-CHL	5	8 (7.3%)

CLI, clindamycin; ERY, erythromycin; LEV, levofloxacin; TE, tetracycline; CHL, chloramphenicol.

### Serotypes, STs, and CCs distribution

**Figure 2:**
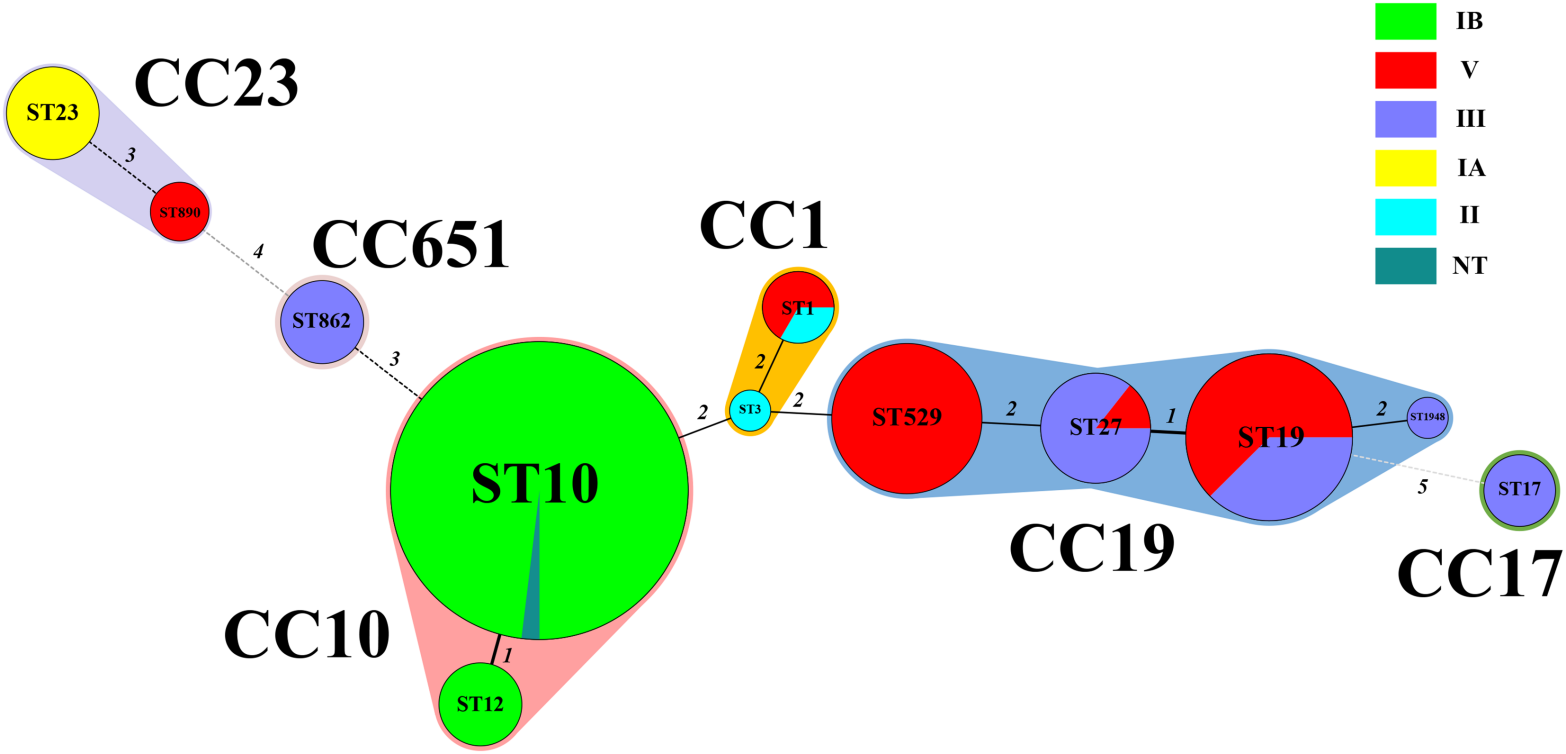
Minimum spanning tree of the 110 *S. agalactiae* strains showing the relationship between ST and cloning complexes (CC) by serotype. Circles represent sequence types (STs); size of each circle indicates the number of strains within the specific type. The serotypes of *S. agalactiae* strains appeared as different colours. Shading denotes STs belonging to the same CC. The numbers between the STs circles indicate the allelic difference between the adjacent STs. CC, clonal complex; ST, sequence type.

Six serotypes were detected in the strains involved in this study: IA, IB, II, III, and V, and one NT. The highest proportion of serotypes was IB (49.1%, 54/110), followed by V (25.5%, 28/110), III (18.2%, 20/110), IA (4.5%, 5/110), and II (1.8%, 2/110). Among the 110 strains, 12 STs were identified, including a new ST type named ST1948 belonging to CC19. Of the 12 STs, the most abundant was ST10 (46.3%, 51/110), followed by ST19 (14.5%, 16/110) and ST529 (11.8%, 13/110). The 12 STs were grouped into six CCs, and the majority of strains (50%, 55/110) belonged to CC10, followed by CC19 (3.6%, 37/110). Except for one strain in CC10, all other CC10 strains were serotype IB. All CC17 and CC651 strains were classified as serotype III strains. All CC19 were serotype V and III strains, and all CC23 were serotype IA and V strains. The MLST minimum spanning tree and serotype distributions are shown in Figure 2.

### Detection of resistance genes and virulence factors

Sixteen antibiotic resistance genes were found in the 110 strains, which primarily mediated the resistance to macrolides, lincosamides, tetracyclines, and chloramphenicol. Details of the antibiotic resistance genes and mutations are presented in Table 2.

**Table 2:** Antibiotic resistance genes or mutations and virulence factors in 110 clindamycin-resistant *Streptococcus agalactiae* strains.

Classes		Percentage of positivity (%)
Antibiotic	Macrolide and Lincosamide	*mreA*(100%), *ermB*(81.8%), *mefA*(15.5%), *msrD*(15.5%), *ermA*(11.8%), *lnuB*(8.2%), *lsaE*(8.2%), *lsaC*(5.5%)
	Tetracycline	*tetM*(25.5%), *tetO*(25.5%), *tetS*(3.6%)
	Chloramphenicol	*cat*(1.8%), *catQ*(7.3%)
	Aminoglycoside	*aph(3′)-III*(16.4%), *ant(6)-Ia*(11.8%), *aac(6′)-aph(2″)*(3.6%)
	Fluoroquinolone	mutations of *gyrA*(S81L) and/or *parC* (S79F or S79Y) (63.6%)
Virulence	Immune evasion	α protein family(100%), *scpB*(1.8%)
	Adhesion	PI(100%), Srr(70%), *pavA*(100%), *lmb*(95.5%), HvgA(2.7%), *fbsA*(1%)
	Tissue damage	*cfb*(100%), *cylE*(100%), *hylB*(34.5%)

All 110 strains harboured the virulence factors *cfb*, *cylE*, *pavA*, and pili*-*related gene clusters, while 105 (95.5%) harboured *lmb*, 74 (67.2%) harboured the Srr1 gene, 58 (52.7%) harboured the Alpha gene, 38 (34.5%) harboured *hylB*, 30 (27.3%) harboured the Rib gene, 19 (17.3%) harboured the ALP1 gene, 3 (2.7%) harboured the HvgA gene, 3 (2.7%) harboured the Alp2-3 gene, 3 (2.7%) harboured the Srr2 gene, and 2 (1.8%) harboured *scpB*. Only one strain contained *fbsA*. Details of the virulence factors are presented in Table 2. Mechanisms of resistance and virulence divided by ST are presented in Supplementary Table S2.

**Figure 3:**
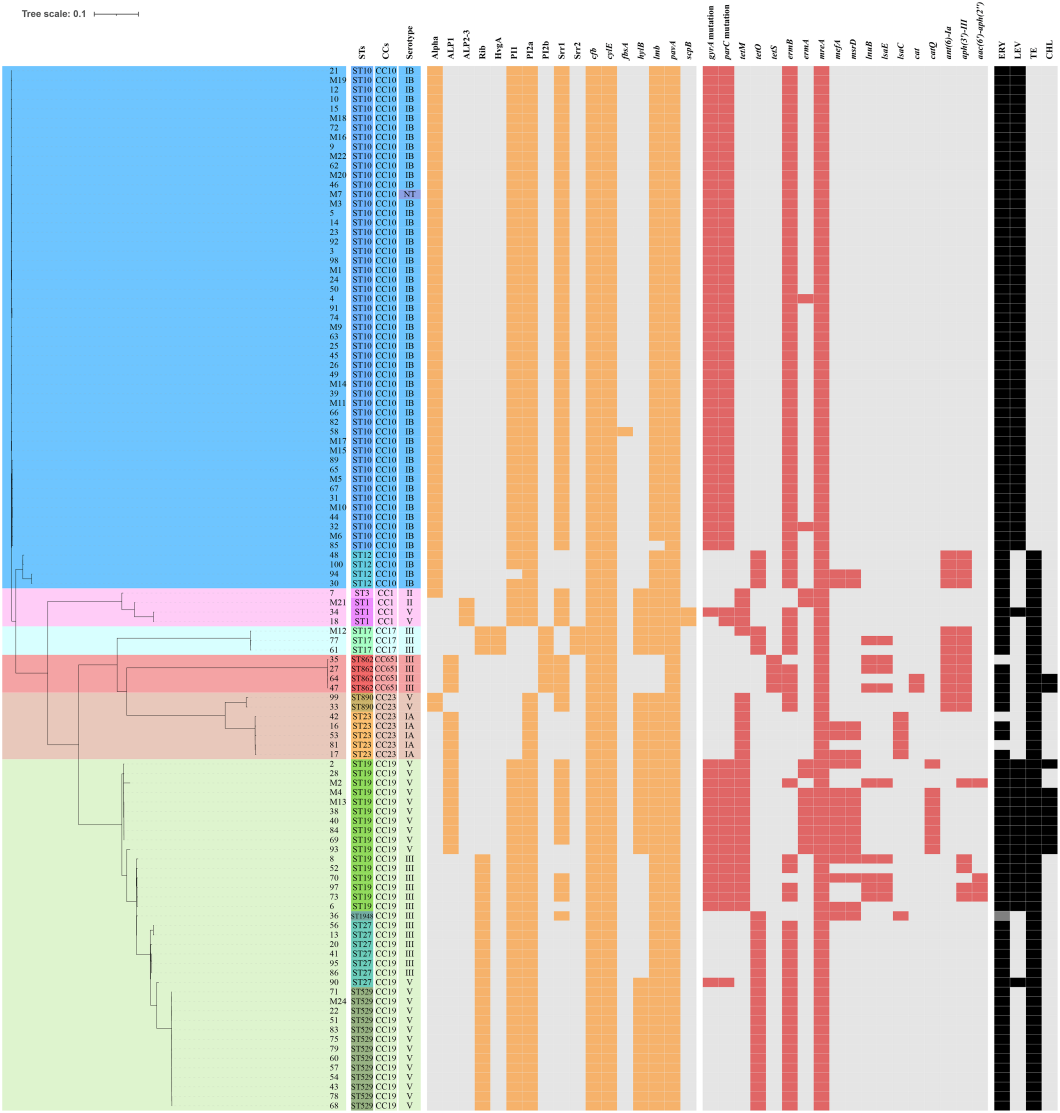
Core genome of the 110 strains of *S. agalactiae.* SNP (Single nucleotide polymorphism) maximum-likelihood phylogenetic tree and heat map showing the virulence and resistance genes. The rectangular block of different colours represents STs, CCs, and serotypes. The spaces are filled in orange or red if the strain harboured the resistance genes or virulence factors, respectively. The rectangular blocks were filled in black when strains had resistant profiles to erythromycin (ERY), levofloxacin (LEV), tetracycline (TE), and chloramphenicol (CHL).

**Figure 4:**
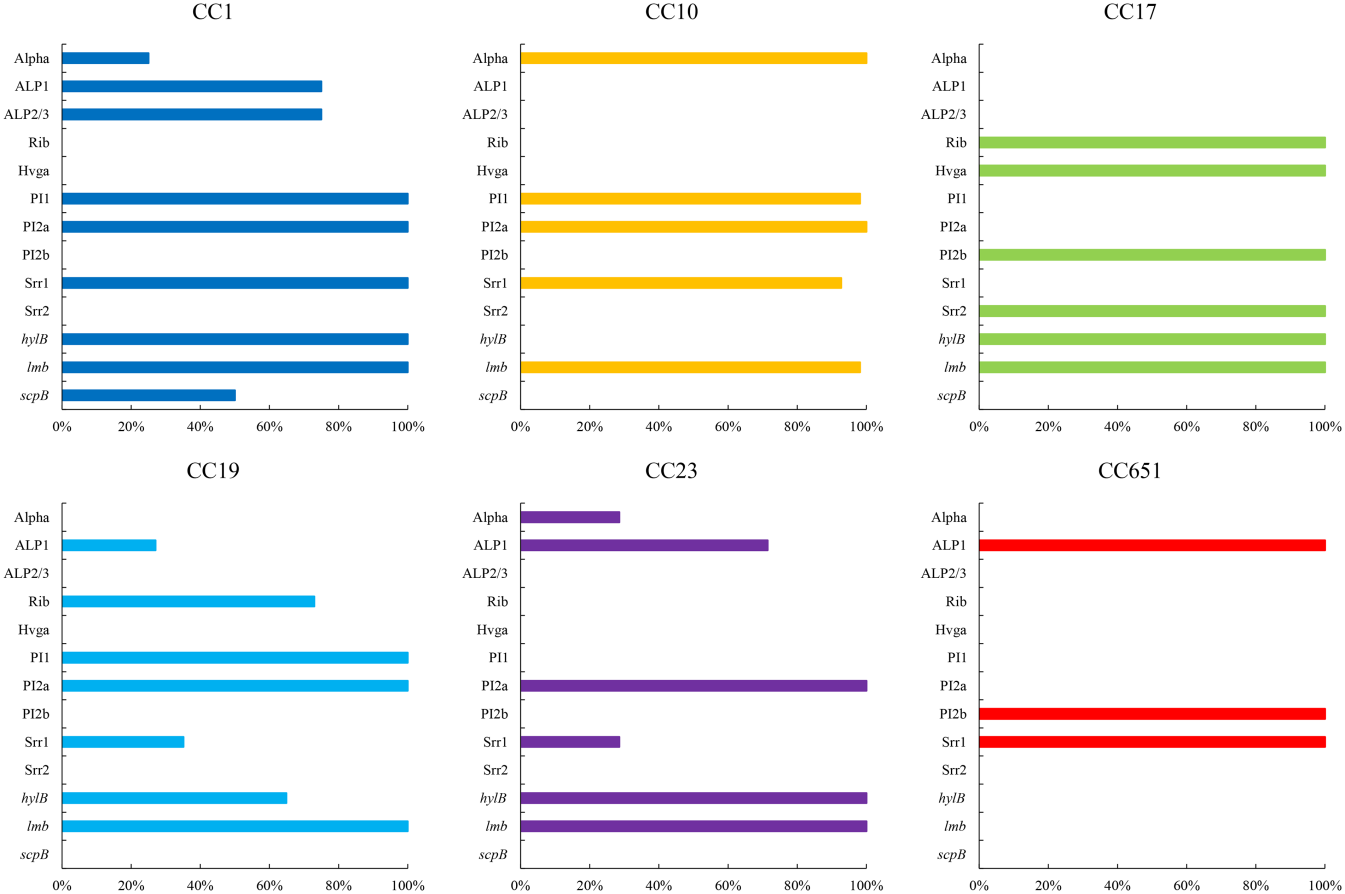
Virulence factors of strains with different CCs (%). Only virulence factors with significant difference among CCs are included (*p* < 0.05).

**Figure 5:**
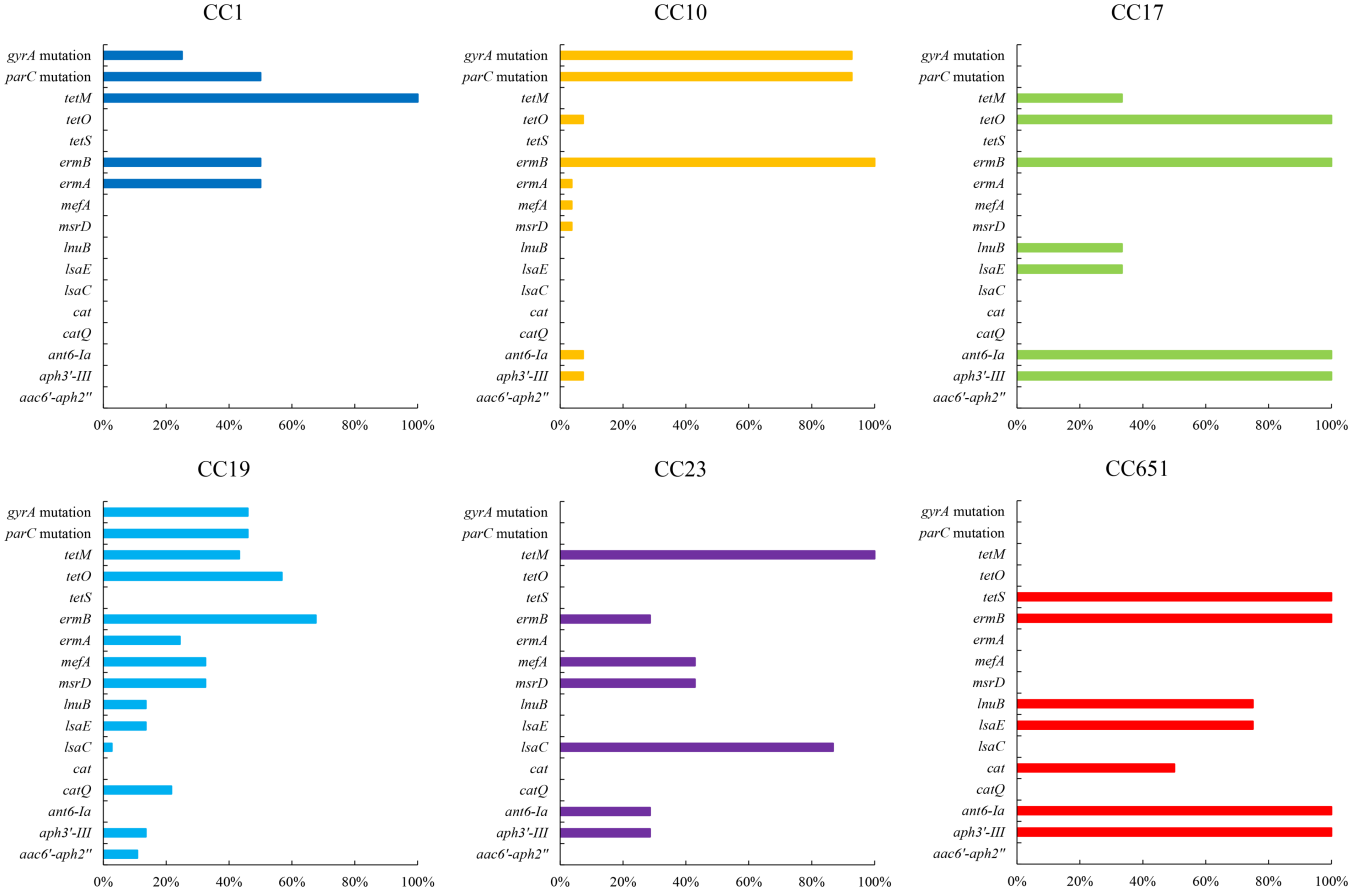
Resistance genes of strains with different CCs (%). Only antibiotic resistance genes with significant difference among CCs are included (*p* < 0.05).

The presence of resistance genes and virulence factors varies in different clonal complexes. In CC10 strains, the mutation incidence of *gyrA* and *parC* was 92.7%, and the carriage rate of virulence factor Alpha was 100%, which was significantly higher than that of other CCs strains (*p* < 0.001). CC17 strains carrying *tetO* was 100%, which was higher than that of the other CCs strains (*p* < 0.001). The carrying rate of the *lsaC* in CC23 strains was 85.7%, which was higher than that of the other CCs strains (*p* < 0.001). The carriage rates of resistance genes *lnuB* and *lsaE*, and the virulence factor ALP1 in CC651 were 75, 75, and 100%, respectively, which were significantly higher than those of the other CCs strains (*p* < 0.001). Furthermore, the virulence factors HvgA and Srr2 are unique to CC17. Virulence factors ALP2-3 and *scpB* are unique to CC1. The resistance gene, *cat* and *tetS,* is unique to CC651. Resistance genes *catQ*, *aac(6′)-aph(2″)* were unique to CC19. Figure 3 shows the core genome SNP maximum-likelihood phylogenetic tree and the resistance gene and virulence factor carrying status. Figures 4, 5 compare the resistance genes and virulence factors of different CCs.

## Discussion

Although *S. agalactiae* remains highly sensitive to penicillin, which is the first-line treatment for the infection, clindamycin is the primary antimicrobial agent for patients with penicillin allergy (ACOG, 2020). However, in China, the high resistance rate of *S. agalactiae* against clindamycin brings formidable challenges to the treatment of patients with penicillin allergy. In this study, we found that clindamycin-resistant strains were resistant to erythromycin, levofloxacin, tetracycline, and chloramphenicol. Furthermore, the proportion of resistant strains with the clindamycin–erythromycin–levofloxacin pattern was 62.7%, and clindamycin–erythromycin–tetracycline–levofloxacin–chloramphenicol-resistant strains were identified. An Australian study found 32% co-resistance of clindamycin and erythromycin in 100 *S. agalactiae* strains (Jones et al., 2022). In Iran, the multiple-resistance rate of *S. agalactiae* was approximately 22% (Motallebirad et al., 2021), while in Brazil, one *S. agalactiae* strain exhibited a multidrug resistance pattern (de Figueiredo et al., 2021a). These studies, including our study, suggest the worsening of clindamycin resistance in *S. agalactiae* globally. In this study, the rates of *S. agalactiae* resistance to clindamycin and erythromycin were substantially higher than those reported in other countries and regions. Thus, more attention is needed for the resistance and multidrug resistance of *S. agalactiae* in China.

Clindamycin and erythromycin resistance mechanisms in *S. agalactiae* are divided into four categories. First, through methylation modification of the ribosome by methyl-transferases, *erm* family genes can confer cross-resistance to clindamycin and erythromycin in strains. Second, the *lsa* and *msr* family genes encoding ABC transporters cause bacterial resistance to clindamycin and erythromycin, respectively. Third, the *mef* and *mre* family of genes encoding antibiotic efflux pumps cause bacterial resistance to erythromycin. Fourth, the *lnu* family genes encoding a nucleotidyl transferase that catalyses the adenylation of clindamycin lead to bacterial resistance to clindamycin (Leclercq, 2002; Wilson, 2016; Dinos, 2017; Vidal et al., 2022). Gene *erm* subtype *ermB* or *ermA*, which is the most important mechanism causing resistance to clindamycin and erythromycin was 91.8% positive of the strains involved in the study, while *mreA* which is a resident gene in *S. agalactiae* (Clarebout et al., 2001) were 100% positive. Among the nine strains that did not carry the *erm* family genes, eight strains carried the *lsa* family genes mediating their resistance to clindamycin, and the remaining strain did not carry any known clindamycin resistance genes, indicating that this strain may have novel mechanisms of antibiotic resistance that warrant further investigation.

*Streptococcus agalactiae* resistance to fluoroquinolones is mainly attributed to mutations in *gyrA* and *parC*. Common mutations in *gyrA* include Ser-81-Leu amino acid substitution, whereas mutations frequently observed in *parC* include Ser-79-Phe or Tyr amino acid substitution (Bob-Manuel et al., 2021; Zhang et al., 2021). In this study, all levofloxacin-resistant strains exhibited *gyrA* and *parC* mutations and were main classified as CC10 strains, except for one strain that harboured *parC* mutation and was susceptible to levofloxacin. This demonstrates that the combination of mutations at *gyrA* position 81 and *parC* position 79 is a crucial factor in fluoroquinolone resistance in *S. agalactiae*. The primary mechanism of chloramphenicol resistance in *S. agalactiae* is that *cat* family genes encode different chloramphenicol acetyltransferases and render the antibiotic inactive (Morici et al., 2017). The strains resistant to chloramphenicol in this study were predominantly CC19, and all carried *catQ* (*n* = 8/110, 7.2%, *p* < 0.001). Simoni et al. found that levofloxacin-resistant *S. agalactiae* strains were also highly resistant to chloramphenicol; however, strains sensitive to levofloxacin did not harbour chloramphenicol resistance gene with unknown relating mechanisms (Simoni et al., 2018). In our study, all *catQ* were detected in levofloxacin-resistant strains, which provided clues to the connection between chloramphenicol and fluoroquinolone resistance.

The main mechanism of tetracycline resistance in *S. agalactiae* is drug efflux mediated by efflux pumps or target protection mediated by ribosomal protection proteins. This study identified three *tet* genes: *tetM*, *tetO*, and *tetS*, all of which encode ribosomal protective proteins (Haenni et al., 2018; Li et al., 2020). The three types of *tet* were distributed differently in different CCs, with *tetM* in CC1, CC17, CC19, and CC23; *tetO* in CC10, CC17, and CC19; and *tetS* in CC651. Furthermore, we found that all the CC1, CC17, and CC651 strains carried *tetM*, *tetO*, and *tetS,* respectively. Except for one strain, which harboured both *tetM* and *tetO*, the other resistant strains contained only one *tet*, indicating that strains with various CCs acquired tetracycline resistance genes independently of one another and that horizontal transfer of *tet* genes between CCs was uncommon.

The cell wall of Gram-positive bacteria is less permeable to aminoglycoside molecules; therefore, *Streptococcus* spp. are intrinsically resistant to aminoglycosides at low concentrations (Krause et al., 2016). In this study, three aminoglycoside resistance genes, *ant(6)-Ia*, *aph(3′)-III*, and *aac(6′)-aph(2″)*, which encode nucleotidyltransferase, phosphotransferase, and acetyltransferase, respectively, inactivated aminoglycosides and increased the aminoglycoside tolerance of the strains’ (Ramirez and Tolmasky, 2010; Krause et al., 2016).

In general, various bacterial strains with multidrug-resistant (MDR) patterns have attracted clinical attention. This study found five MDR patterns among the strains, which were fewer than those identified by Mudzana et al. (2021) but with a similar proportion. The differences in MDR patterns between strains might be influenced by the use of antibiotics in the clinic, which selects MDR strains with resistance genes or mutations. As a result, we advocate monitoring common antibiotics for the treatment and prophylaxis of *S. agalactiae* to predict the future development of antibiotic resistance.

The virulence factors of *S. agalactiae* determine its colonisation, persistence to immune attacks, translocation, and modes of invasion, resulting in different levels of pathogenicity. In this study, the most frequently detected α protein was Alpha, which was most commonly correlated with CC10. But Alp1 was the main α protein in a Nigerian study (Bob-Manuel et al., 2021). All strains expressed pili. PI1 and PI2a were mainly detected in CC10. PI2b was detected in CC17 and CC651 strains. The combination of PI1 and PI2a was the most common. CC17, which is associated with high virulence and pathogenicity in infants (Deshayes et al., 2021) carried only PI2b. HvgA is the most characteristic virulence factor of *S. agalactiae*, and its expression enhances adhesion to intestinal epithelial cells, choroidal epithelial cells, and microvascular endothelial cells that constitute the blood–brain barrier (BBB; Landwehr-Kenzel and Henneke, 2014). All three strains carrying HvgA in this study were classified in CC17. Srr1 and Srr2 contribute to *S. agalactiae* colonisation through a latch mechanism (Wang et al., 2014; Lannes-Costa et al., 2021). Srr1 showed the highest carriage in CC10 like Alpha. Srr2 is exclusive in CC17, and it has been associated with adhesion and invasion of brain endothelial cells and highly toxic meningitis (Deshayes et al., 2021).

In conclusion, clindamycin-resistant *S. agalactiae* showed polymorphism in molecular type and serotype, even serotype associated with high virulence. Furthermore, multiple resistance to erythromycin, levofloxacin, tetracycline, and chloramphenicol was observed. *S. agalactiae-*infected patients with penicillin allergy in China have fewer antibiotic options because of the rapid rise in *S. agalactiae* resistance. Vancomycin remains effective against *S. agalactiae* resistant to second-line antibiotics (ACOG, 2020). The data in this study will provide a basis for clinical monitoring of antibiotic resistance in *S. agalactiae*, as well as the evaluation, prevention, control, and treatment of *S. agalactiae* infections.

## Data availability statement

The datasets presented in this study can be found in online repositories. The names of the repository/repositories and accession number(s) can be found at: https://nmdc.cn/, NMDC10018268.

## Author contributions

ZL, AD, and JuanL conceived the study and designed the experimental procedures. ZL and XJ performed experiments. ZL, XJ, and JieL analysed the data. ZL prepared and generated the tables and figures accompanying the manuscript. AD, JuanL, WJ, HZ, XG, BM, SM, and CC critically reviewed the manuscript. LD, QS, XH, and PG contributed the reagents and materials. All authors have contributed to the manuscript and approved the submitted version.

## Funding

This study was supported by the National Natural Science Foundation of China (81861138053).

## Conflict of interest

The authors declare that the research was conducted in the absence of any commercial or financial relationships that could be construed as a potential conflict of interest.

## Publisher’s note

All claims expressed in this article are solely those of the authors and do not necessarily represent those of their affiliated organizations, or those of the publisher, the editors and the reviewers. Any product that may be evaluated in this article, or claim that may be made by its manufacturer, is not guaranteed or endorsed by the publisher.

## Footnotes

^1^ https://pubmlst.org/

^2^ https://cge.food.dtu.dk/services/ResFinder/
